# Modification of Prednisolone Acetate Release from Monolithic 3D-Printed Systems: The Role of Formulation Composition and Process Parameters

**DOI:** 10.3390/pharmaceutics18070793

**Published:** 2026-06-28

**Authors:** Aleksandra Ćoškov, Nemanja Todorović, Maja Buljčik Čupić, Miluša Vranka, Luka Jolić, Nataša Milošević, Mladena Lalić-Popović

**Affiliations:** 1 Department of Pharmacy, Faculty of Medicine Novi Sad, University of Novi Sad, 21000 Novi Sad, Serbia; aleksandra.coskov@mf.uns.ac.rs (A.Ć.); milusavranka@gmail.com (M.V.); 202060@mf.uns.ac.rs (L.J.); natasa.milosevic@mf.uns.ac.rs (N.M.); mladena.lalic-popovic@mf.uns.ac.rs (M.L.-P.); 2 Department of Otorhinolaryngology, Faculty of Medicine Novi Sad, University of Novi Sad, 21000 Novi Sad, Serbia; maja.buljcik-cupic@mf.uns.ac.rs; 3 Clinic for Otorhinolaryngology and Head and Neck Surgery, University Clinical Center of Vojvodina, 21000 Novi Sad, Serbia; 4 Centre for Medical and Pharmaceutical Investigations and Quality Control (CEMPhIC), Faculty of Medicine Novi Sad, University of Novi Sad, 21000 Novi Sad, Serbia

**Keywords:** 3D printing, FDM, HME, prednisolone acetate, infill density, sodium alginate

## Abstract

**Background/Objectives**: A major advantage of 3D printing technology is the ability to modify drug release by adjusting formulation composition and printing parameters. The aim of this study was to develop and characterize 3D-printed tablets containing prednisolone acetate and to investigate the effects of formulation composition and printing parameters, namely infill density and pattern, on the drug release profile. **Methods:** Filaments composed of polyvinyl alcohol, sorbitol, and prednisolone acetate, with sodium alginate incorporated in selected formulations, were prepared using hot melt extrusion. The obtained filaments were characterized and used for the fabrication of tablets via fused deposition modeling. The resulting tablets were evaluated in terms of mass variation, dimensions, hardness, content uniformity and drug release rate. **Results:** The extrusion of polyvinyl alcohol and prednisolone acetate in the absence of additional excipients resulted in a defective filament, highlighting the need for sorbitol incorporation. In contrast, all other filament formulations (F2–F4) exhibited a uniform structure and homogeneous drug distribution. The 3D-printed tablets complied with pharmacopeial requirements for mass variation and content uniformity and demonstrated good precision and reproducibility in terms of dimensions and hardness. Lower infill density was associated with faster drug release, while the presence of sodium alginate resulted in slower release, particularly at higher infill percentages and with a gyroid infill pattern. Furthermore, formulations with higher sorbitol content demonstrated an increased release rate of prednisolone acetate. **Conclusions:** Infill density was identified as the dominant factor affecting release kinetics. Among the tested formulations, A100G (gyroid structure with 100% infill density), containing prednisolone acetate, polyvinyl alcohol, sorbitol, and sodium alginate, proved most suitable for achieving sustained drug release.

## 1. Introduction

Corticosteroids are a group of drugs often used in clinical practice, with immunomodulatory, anti-inflammatory and antineoplastic effects [1,2,3]. In modern medicine, the term corticosteroids mainly refers to agents with glucocorticoid activity, including representatives such as prednisolone, prednisone, dexamethasone, hydrocortisone and others [1,3,4]. Owing to their broad spectrum of therapeutic applications and well-established pharmacological efficacy, corticosteroids are often used in the treatment of various conditions, and their administration requires careful dosing and supervision. Although the existence of conventional formulations of corticosteroids significantly facilitates the implementation of therapy, large inter-individual differences among patients contribute to the inadequacy of the dose, as well as the risk of side effects. Although monolithic 3D-printed systems cannot eliminate physiological sources of variability, they may help reduce formulation-related variability by enabling precise dose administration, tailored drug release profiles, and the possibility of personalized therapy according to individual patient needs.

Contemporary approaches to overcoming the limitations of conventional pharmaceutical dosage forms include innovative technologies such as three-dimensional (3D) printing [5,6]. The possibility of individualizing therapy, combining several active pharmaceutical substances (API) within one pharmaceutical form, precise dosing, and the possibility of modifying the release of API by adjusting not only the composition of the formulation but also the printing parameters are just some of the numerous advantages of this technology [5,7]. One of the most commonly used 3D printing techniques is fused deposition modeling (FDM) due to its simplicity, economy, availability, and the large number of excipients suitable for the formulation of various 3D-printed pharmaceutical forms [7,8,9]. FDM is based on a layer-by-layer printing process, where the molten filament with API is extruded through a heated nozzle onto the work plate, forming an object of the desired shape and size [7].

To obtain the filament, which serves as an intermediate product in 3D printing, the hot melt extrusion (HME) technique is commonly employed. HME is a process that creates a solid, amorphous dispersion of API in a polymer matrix by stirring and heating, without the use of solvents [10]. Extrusion temperature, the selection of suitable excipients, and the choice of API are among the key factors that directly influence filament extrudability [11,12,13]. In addition to the composition of the formulation, processing parameters, such as infill density and infill pattern, play an important role in defining the porosity and internal structure of the system. This may be of particular importance for poorly soluble APIs, which may further influence their release kinetics [14,15].

The thermoplastic polymer, as the major component of the formulation, forms the basis for a drug–polymer matrix suitable for successful 3D printing. The most commonly used polymers of pharmacopeial quality in the FDM technique for 3D printing are polyvinyl alcohol (PVA), polyvinyl pyrrolidone (PVP) and polylactic acid (PLA) [12,16]. PVA is a hydrophilic, biodegradable and biocompatible polymer widely used in FDM printing [17,18,19]. The hydroxyl groups in the PVA structure allow it to swell upon contact with water, while the dissolution behavior of PVA itself can be modified by varying the geometry and internal structure of the 3D-printed objects [17,18]. It is important to note that PVA is highly hygroscopic and has limited mechanical strength compared to other thermoplastic polymers. However, despite these limitations, its characteristics make it a suitable material for this research. In order to increase the flexibility of the filament and facilitate the process of extrusion and printing, various plasticizers (polyethylene glycol, sorbitol) can be added to the formulation [16]. Additionally, hydrophilic polymers, such as sodium alginate, may be employed to modify API release by promoting the formation of a matrix capable of swelling upon contact with the medium, thereby enabling controlled drug release [20]. Sodium alginate is a natural, non-toxic polysaccharide that has attracted interest in 3D printing applications [12,21]. Its favorable physicochemical properties, including biocompatibility and gel-forming ability, make it a promising excipient for the design of advanced drug delivery systems with tunable release-modifying characteristics [20,22]. Some limitations of sodium alginate include its high hydrophilicity and relatively poor mechanical strength, which is why it is frequently combined with other polymers [23]. Therefore, a combination of sodium alginate with thermoplastic carriers such as PVA may be advantageous, as PVA can provide the required processability and filament-forming properties, while sodium alginate contributes to the desired release-modifying function.

Prednisolone acetate (PAC) is a prodrug and an esterified form of prednisolone with an acetate group on the C21 atom [24]. Through acetylation of the prednisolone molecule, solubility is reduced, whereas stability and lipophilicity are increased. Given these characteristics of PAC, the formulation of this drug in conventional pharmaceutical forms can be challenging in terms of achieving optimal bioavailability and modified release. In this context, the application of FDM 3D printing, in combination with HME, represents a promising approach for the development of personalized pharmaceutical dosage forms with modified release.

The aims of this study were to develop and characterize five different filaments for 3D printing, including four formulations containing PAC and one placebo formulation without PAC, prepared via HME technology using selected formulation components such as PVA, sorbitol, and sodium alginate. Furthermore, FDM 3D printing was applied to produce twelve different PAC tablet formulations from the selected filaments, followed by their physicochemical characterization. In addition, the influence of formulation composition, as well as 3D processing parameters, namely infill density and infill pattern, on the release rate of PAC from the printed tablets was evaluated.

## 2. Materials and Methods

### 2.1. Materials

PAC (Farmalabor, Canosa di Puglia, Italy) was used as the API in this research work. Sorbitol (S, Parteck^®^ SI 150, Merck, Darmstadt, Germany) was employed as a plasticizer. Sodium alginate (M = 300,000–350,000 g/mol, Carl Roth, Karlsruhe, Germany) was used as a hydrophilic polymer, while partially hydrolyzed PVA (Parteck^®^ MXP, Merck, Germany) was used as a thermoplastic polymer material. Testing the release rate of PAC from 3D-printed tablets was conducted in an aqueous solution of 0.5% sodium lauryl sulfate (Lachner, Neratovice, Czech Republic) to ensure adequate drug solubility, sink conditions, and analytical reliability during dissolution testing.

### 2.2. Methods

#### 2.2.1. Sieve Analysis

Sieve analysis was performed in triplicate using a vibratory sieve shaker (Retsch, Haan, Germany). In accordance with the requirements of the European Pharmacopoeia (Eur. Ph., Chapter 2.9.34), sieves with aperture sizes of 90 μm, 125 μm, 180 μm, 250 μm, and 355 μm were used for the analysis [25]. The particle size distribution of PAC, sorbitol, sodium alginate, and PVA was evaluated.

A 25 g sample of each material was placed on the sieve with the largest aperture size and sieved continuously for 5 min at an amplitude of 1 mm (confirmed end point of sieving). Sieve analysis was used to assess the potential for powder blend segregation during the extrusion process due to screw rotation during filament production, and the results are presented graphically.

#### 2.2.2. Preparation of Powder Formulations

Five powder formulations (F0, F1, F2, F3 and F4) were prepared. Formulation F0 was the placebo formulation, containing only PVA and sorbitol. Formulation F1 contained PAC and PVA. Besides these components, formulations F2, F3 and F4 also contained sorbitol, while sodium alginate was additionally included in formulation F4. The composition of the prepared formulations is presented in Table 1. Formulation development was performed stepwise, with modifications in polymer and excipient ratios being introduced based on the performance of the preceding formulation during HME processing, FDM printing and drug release testing.

The proportion of sorbitol was determined according to the manufacturer’s recommendation for Parteck^®^ MXP [10]. Powder blending was carried out using a laboratory tumble mixer (Farmalabor, Canosa di Puglia, Italy), ensuring adequate homogeneity of the mixtures.

#### 2.2.3. Loss on Drying

Loss on drying was determined in triplicate using a moisture analyzer based on the thermogravimetric principle (MA210.R Moisture Analyzer, Radwag, Radom, Poland). The sample was heated to 105 °C, while the change in mass was automatically recorded until a constant mass was achieved. Loss on drying was expressed as the percentage change in mass before and after completion of the drying process. The analysis was performed for PAC, sorbitol, sodium alginate, PVA, and the powder formulations (before and after drying for 24 h at 40 °C).

#### 2.2.4. Determination of Prednisolone Acetate Content in Powder Blends Prior to Extrusion

Determination of PAC content in powder blends was performed in triplicate after 24 h of drying and prior to extrusion. Samples of all powder formulations weighing approximately 0.2 g were collected and dissolved in 100 mL of 0.5% sodium lauryl sulfate solution. The test was performed in triplicate. Each volumetric flask was placed in an ultrasonic bath (Bandelin, Berlin, Germany) for 15 min, after which the samples were filtered through a 0.45 µm membrane filter (Isolab, Eschau, Germany).

The content was determined using a UV/Vis spectrophotometer (Agilent 8453, Agilent Technologies, Santa Clara, CA, USA) at a wavelength of 248 nm, according to the recommendation of the PAC monograph in Ph. Eur. [25]. The calibration curve was recorded over the concentration range of 3.125–50 µg/mL and showed linearity (R^2^ = 0.9986).

The content was expressed as a percentage relative to the declared (theoretical) value, and the results are presented in tabular form.

#### 2.2.5. Hot Melt Extrusion

Filaments were prepared from the corresponding powder formulations (F0–F4) using the HME technique and a single-screw extruder (Noztek Touch, Shoreham-by-Sea, UK). Suitable filament characteristics, including low roughness, reduced brittleness, and minimal waviness, were achieved by adjusting the extrusion temperature and screw rotation speed.

The placebo formulation was used to optimize the extrusion parameters, after which the PAC-containing formulations were extruded. The screw rotation speed was first set to 30 rpm, while the extrusion temperature was tested within the range of 130–180 °C (heater T1/T2). The screw speed for formulations F0, F1, and F2 was set at 30 rpm, whereas for formulations F3 and F4 it was increased to 40 rpm. Formulation F1 was processed at 180–185 °C (heater zones T1/T2), while formulations F3 and F4 were processed at 150–155 °C (T1/T2). A slightly lower second-zone temperature was applied for F2 (150–145 °C). The extrusion temperatures for all formulations were applied according to the manufacturer’s recommendation for Parteck^®^ MXP [10].

#### 2.2.6. Characterization of Extruded Filaments

The quality of the manufactured filaments was assessed by visual inspection of color, tortuosity, surface roughness and flexibility. Filaments were imaged using a binocular loupe (Zeiss Stemi 508, Carl Zeiss, Oberkochen, Germany) with an accompanying digital camera (Zeiss Axiocam ERC 5s, Carl Zeiss, Germany). Filament F1 did not show satisfactory characteristics required for printing and was excluded from further tests. Accordingly, characterization of the filament in terms of the quantitative composition of PAC and the tablet printing process was performed exclusively on the remaining formulations (F2, F3 and F4).

For the determination of PAC content in the filaments, samples weighing approximately 0.2 g were taken from three randomly selected locations and dissolved in 100 mL of 0.5% sodium lauryl sulfate solution. The filament was disintegrated under the influence of ultrasound, after which the samples were prepared and analyzed using a UV/Vis spectrophotometer (Agilent 8453, Santa Clara, USA) according to the same method described in Section 2.2.4.

#### 2.2.7. Fourier-Transform Infrared Spectroscopy

Fourier-transform infrared (FTIR) spectroscopy was performed for pure PAC as well as for filaments prepared from formulations F2–F4. The analysis was carried out using an FTIR spectrophotometer (Nicolet IS10, Thermo Scientific, Waltham, MA, USA), while data acquisition was performed using Omnic 8.1 software (Thermo Scientific, USA). A background spectrum was recorded prior to the analysis of each sample. Spectra were collected in the wavenumber range of 4000–400 cm^−1^, with 32 scans per sample at a resolution of 4 cm^−1^. The results of the analysis were presented graphically.

#### 2.2.8. X-Ray Powder Diffraction

X-ray powder diffraction (XRPD) was used to characterize pure PAC and filaments prepared from formulations F2, F3, and F4. The analysis was performed using a powder diffractometer (MiniFlex 600, Rigaku, Tokyo, Japan) with monochromatic CuKα radiation. Measurements were carried out over a 2θ range of 5–75°, with a step size of 0.05° and an acquisition time of 5 s per step, under operating conditions of 40 kV and 15 mA. The obtained diffractograms were graphically processed and presented using FullProf Suite software (version June 2026).

#### 2.2.9. Production of Tablets by 3D Printing

FDM 3D printing technology was used to produce 3D-printed tablets (printlets). Printing was performed using the Ultimaker S3 device (Ultimaker, Copenhagen, The Netherlands). The cylinder model was saved as a .stl file and loaded into the Ultimaker Cura program (v4.8.0) for printing preparation.

In the software, printing parameters (including temperature and printing speed) were adjusted, while the infill density and infill pattern of the printlets were varied. The printed object was cylindrical, with either a lines or gyroid infill pattern, while the infill density was set at 50% or 100%. Tablet dimensions were adjusted to maintain a constant diameter for all tablets (10 mm), whereas the height was varied depending on formulation composition and infill density in order to achieve the target mass of 0.2 g and the therapeutic PAC dose of 10 mg. Data on infill density, infill pattern, and printlet dimensions are presented in Table 2. Printlets were labeled according to formulation composition and printing parameters, where S denotes formulations obtained from filament F2 with higher (45%) sorbitol content, RS denotes formulations prepared from F3 with reduced sorbitol content (25%), and A denotes formulations produced from filament F4 containing sodium alginate. Numbers indicate infill density (%), while L and G denote line and gyroid infill patterns, respectively.

**Figure 1:**
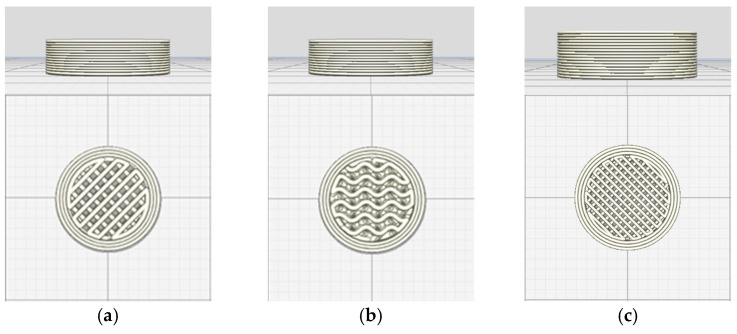

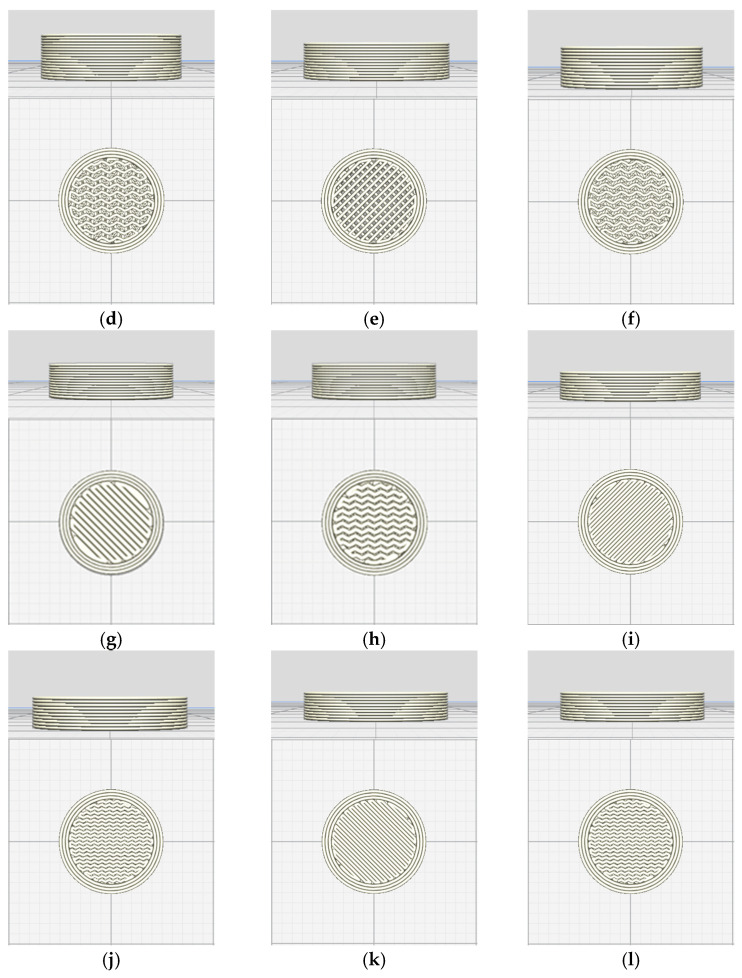
Schematic representations of the 3D objects with 50% infill, (**a**) S50L, (**b**) S50G, (**c**) RS50L, (**d**) RS50G, (**e**) A50L, and (**f**) A50G, and of the 3D objects with 100% infill, (**g**) S100L, (**h**) S100G, (**i**) RS100L, (**j**) RS100G, (**k**) A100L, and (**l**) A100G.

Tablets were printed using a BB 0.4 brass nozzle. The nozzle temperature was varied starting from the temperature previously established for extrusion (145 °C), beginning at the same value and then increasing in 5 °C increments until satisfactory printing was achieved. The optimal nozzle temperature for filament F2 was 160 °C, with the first layer printed at the same temperature. The optimal printing temperature was determined to be 170 °C for filaments F3 and F4, while the temperature of the initial (first) layer was 175 °C. The build plate temperature was set at 60 °C. The printing speed was 15 mm/s. Schematic representations of the printlet models are shown in Figure 1.

#### 2.2.10. Characterization of Printlets

##### Mass Variation

Mass variation was evaluated according to the recommendations of Eur. Ph. 12, with certain modifications [25]. A sample of ten tablets was weighed individually using an analytical balance, and the mean mass was subsequently calculated. Mass variation was expressed as the percentage deviation from the mean value. According to pharmacopeial requirements, the permitted deviation for tablets weighing 0.2 g is ±7.5%, with no more than two individual masses allowed to fall outside this range. At the same time, no individual mass may deviate by more than ±15% from the mean mass.

##### Uniformity of Content

Content uniformity was evaluated using a sample of 10 printlets, as recommended by Eur. Ph. 12 for solid dosage forms. According to the pharmacopoeia, Test B is applied for tablets, and the content may deviate by ±15% from the mean content value [25].

PAC content was determined using the same sample preparation and measurement procedure described in Section 2.2.4. Content was expressed as a percentage relative to the mean measured content of the 10 samples. The results are presented graphically.

##### Dimensions and Hardness of Printlets

The dimensions of the 10 obtained printlets from each formulation, including height and diameter, were measured using a vernier caliper, expressed in millimeters, and presented in tabular form.

The hardness of ten 3D-printed tablets from each formulation was measured centrally and laterally from the upper surface of the tablet using an indentation test with a durometer (PCE Instruments, Meschede, Germany) equipped with the Shore A scale (denoted as hardness A). The test was performed by placing the durometer tip on either the central or lateral part of the tablet and pressing it against the tablet surface, whereby the resistance of the material was recorded. Hardness values were read directly from the durometer and presented in tabular form.

##### Drug Release Rate

The drug release study was performed according to pharmacopeial recommendations for this type of pharmaceutical dosage form, using Apparatus 2 (paddle apparatus) [25]. The paddle rotation speed was set at 50 rpm, and 0.5% sodium lauryl sulfate solution was used as the dissolution medium. The medium volume was 900 mL, and the test was conducted in triplicate.

Aliquots of 5 mL were withdrawn at predetermined time intervals of 3, 7, 10, 15, 20, 25, 30, 45, 60, 90, 120, 150, 180, 210, and 240 min. PAC concentration was measured using the same method described in Section 2.2.4.

##### Statistical Analysis

Statistical analysis of the data was performed using a model-independent approach for the comparison of formulations exhibiting similar sustained-release dissolution profiles. The difference factor (f_1_) and similarity factor (f2) were calculated using the *DD Solver* add-in for Microsoft Excel. Dissolution profiles were considered similar for f_1_ values lower than 15 and f_2_ values higher than 50 [26].

Kinetic modeling of dissolution profiles was performed only for the formulations exhibiting the fastest and the slowest release in order to compare their release kinetics. The following models were applied: Higuchi, Weibull, Hixson–Crowell, Korsmeyer–Peppas, Gompertz 1 and 2, and Logistic 1 and 2. The model showing the best fit to the PAC dissolution profile was characterized by the highest R^2^ value, the lowest Akaike Information Criterion (AIC), and the highest Model Selection Criterion (MSC).

## 3. Results

### 3.1. Sieve Analysis

**Figure 2:**
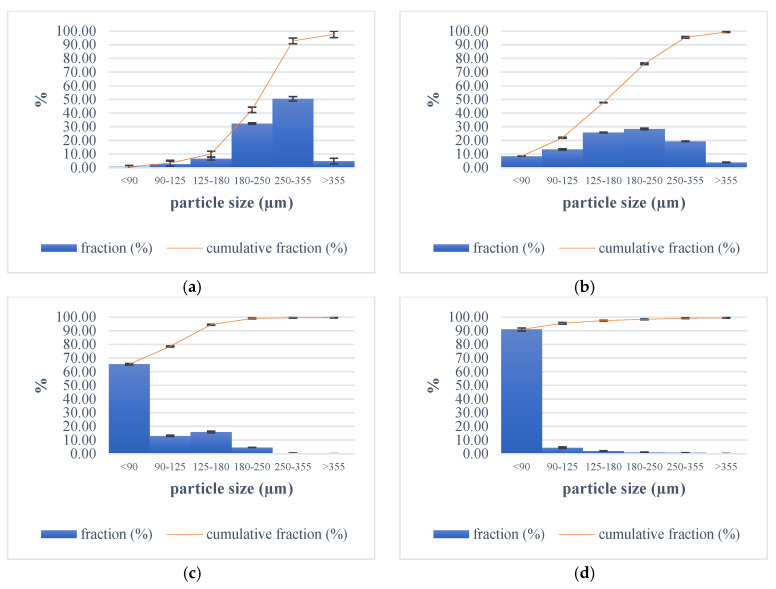
Particle size distribution obtained by sieve analysis: (**a**) PAC; (**b**) sorbitol; (**c**) sodium alginate; (**d**) PVA.

Figure 2a–d show the results of the sieve analysis of the initial powder components. The sieve analysis results demonstrated that the PAC powder had the largest particles, while the smallest particles were observed in the PVA powder. Sorbitol powder showed the widest particle size distribution, whereas PVA powder exhibited the narrowest distribution.

### 3.2. Loss on Drying

**Figure 3:**
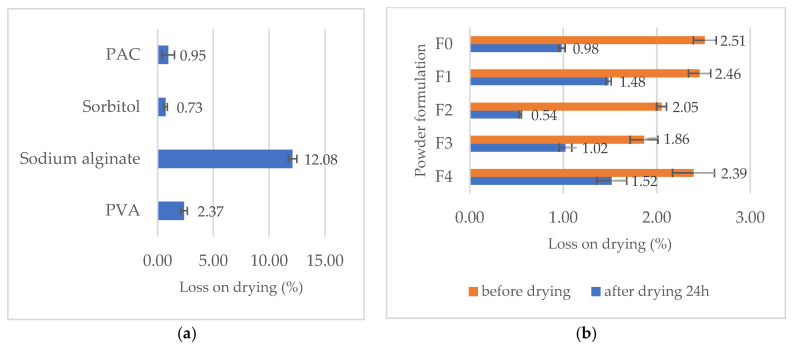
Loss on drying (%) of the investigated samples: (**a**) API and excipients; (**b**) powder formulations.

The results from loss on drying testing are shown in Figure 3a,b. The highest loss on drying was observed in sodium alginate (12.08%), while the PVA sample showed a loss of 2.37%. Moisture can adversely affect the extrudability of materials containing PVA. After drying at 40 °C for 24 h, all powder formulations exhibited loss on drying values below 2%.

### 3.3. Determination of Prednisolone Acetate Content in Powder Blends Prior to Extrusion

The results of PAC content determination in powder formulations are presented in Table 3. The obtained results indicate that all powder formulations complied with the specified acceptance criterion, allowing for a deviation of ±5% from the declared value. The low standard deviation values indicate good content uniformity of the formulations and confirm the suitability of single-screw extrusion for formulation preparation.

### 3.4. Characterization of Extruded Filaments

**Figure 4:**
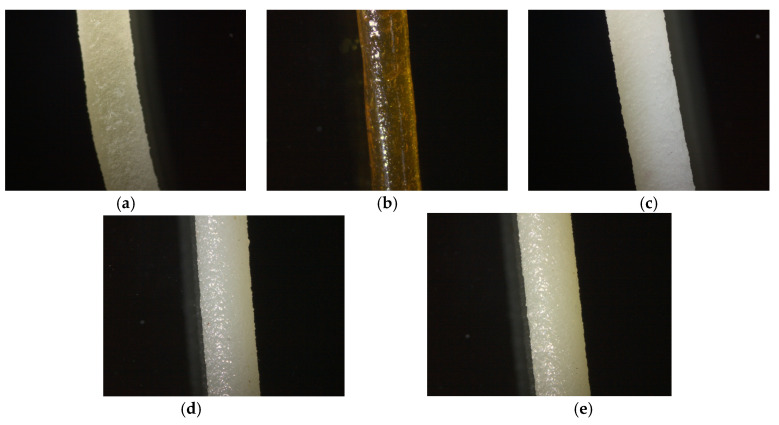
Appearance of prepared filaments: (**a**) F0; (**b**) F1; (**c**) F2; (**d**) F3; (**e**) F4.

The appearance of filaments prepared from powder formulations F0–F4 is shown in Figure 4. First, a noticeable difference in filament color was observed. The placebo filament (F0) exhibited a less uniform morphology and a rougher surface texture compared to the other formulations. The filament prepared from the formulation containing only PAC and PVA (F1) was transparent, dark brown, highly smooth, and uneven in thickness, with visible air bubbles. In contrast, filaments F2 and F3 were lighter in color compared to F4. The filament containing sodium alginate (F4) had a slightly yellowish appearance. Filaments F2, F3 and F4 were opaque, uniform in thickness, and exhibited very similar textures.

The results of PAC content determination in the filaments showed a content of 96.89± 1.41% for formulation F2, 97.97 ± 1.85% for formulation F3 and 95.84 ± 0.62% for formulation F4. The obtained values were in accordance with the expected results and were comparable to those obtained for the corresponding powder formulations, indicating that no significant PAC loss occurred during the extrusion process.

### 3.5. Fourier-Transform Infrared Spectroscopy

**Figure 5:**
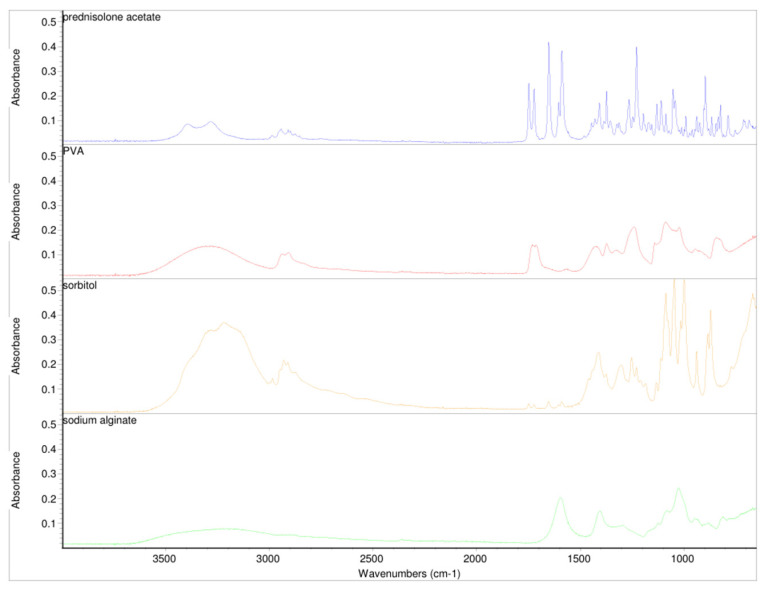
FTIR spectra of pure components.

**Figure 6:**
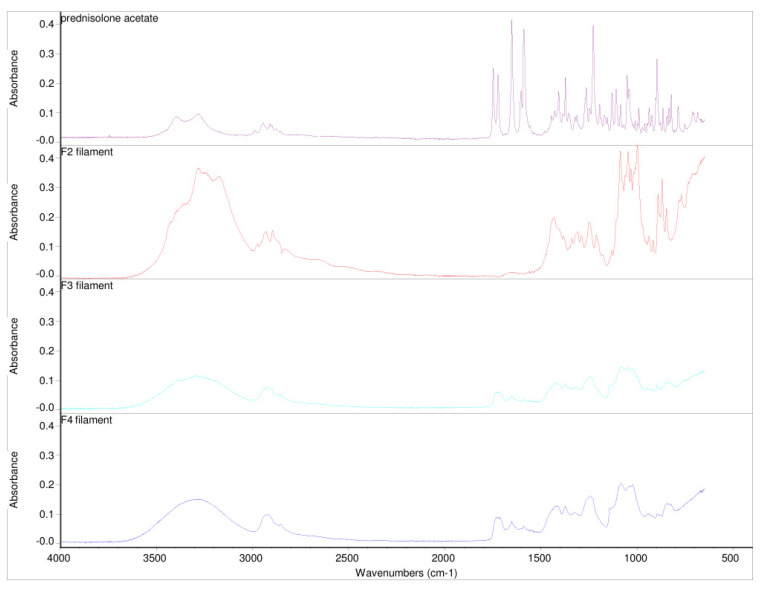
FTIR spectra of pure PAC and extruded filaments F2, F3 and F4.

The results of FTIR analysis of pure components and extruded formulations are presented in Figure 5 and Figure 6. PAC exhibited characteristic absorption bands in the regions around 3400 cm^−1^ and 2900 cm^−1^, with intense bands in the 1750–1700 cm^−1^ and 1650–1600 cm^−1^ regions. Additional distinct peaks were observed below 1500 cm^−1^. PVA showed a broad absorption band between 3600 and 3200 cm^−1^ and absorptions in the regions around 2900 cm^−1^ and 1600–1500 cm^−1^. Sorbitol displayed a broad absorption band in 3600–3000 cm^−1^, a band near the 2900 cm^−1^ region and multiple intense peaks in the fingerprint region. The FTIR spectrum of sodium alginate was characterized by a broad absorption band and several distinct bands located around 1600 cm^−1^, 1400 cm^−1^, and within the 1100–1000 cm^−1^ region.

The FTIR spectra of pure PAC as well as extruded formulations (F2–F4) are shown in Figure 6. In all formulations, a broad absorption band in the area of 3600–3200 cm^−1^ as well as several bands in the areas around 1600–1400 cm^−1^ and 1100–1000 cm^−1^ are observed. The dominance of the absorption bands of the matrix-forming components is observed, while the PAC bands are weakly visible. Additionally, the PAC bands in the fingerprint area are of lower intensity. What is significant is that no new absorption bands were formed in this analyzed spectral range.

### 3.6. X-Ray Powder Diffraction

**Figure 7:**
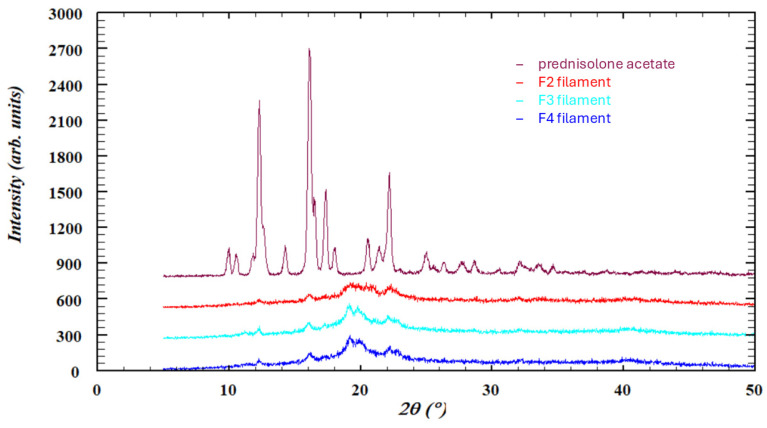
XRPD diffractograms of pure PAC and filament formulations F2, F3 and F4.

The physical state of the pure PAC, as well as in the filaments, was assessed using XRPD (Figure 7). The diffractogram shows numerous sharp and intense peaks, with the most prominent reflections around 2θ = 12.5° and 16°, along with additional peaks in the 10–22° region and several reflections of lower intensity up to approximately 40° 2θ. On the other hand, analysis of the diffractograms of the filament formulations F2, F3 and F4 revealed a broad halo with a maximum at approximately 2θ = 19–20°. The characteristic sharp diffraction peaks of PAC, especially those at approximately 12.5° and 16° 2θ, were not observed in the diffractograms of the filaments.

### 3.7. Production of Tablets by 3D Printing

**Figure 8:**
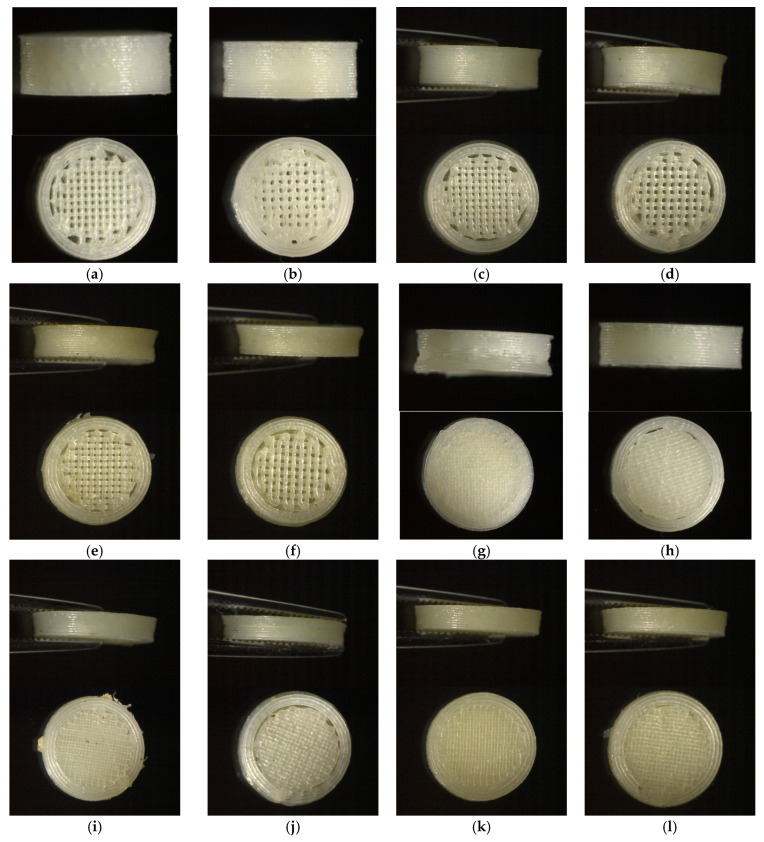
Frontal and lateral images of printlets with 50% infill, (**a**) S50L, (**b**) S50G, (**c**) RS50L, (**d**) RS50G, (**e**) A50L, and (**f**) A50G, and printlets with 100% infill, (**g**) S100L, (**h**) S100G, (**i**) RS100L, (**j**) RS100G, (**k**) A100L, and (**l**) A100G.

Figure 8 shows the prepared 3D-printed tablets obtained from filament formulations F2, F3 and F4. Frontal and lateral views of the tablets are presented. A slight difference in tablet color was observed, with tablets prepared from formulations containing sodium alginate exhibiting a more pronounced yellowish tint, consistent with the appearance of the corresponding filament.

### 3.8. Characterization of Printlets

#### 3.8.1. Mass Variation

**Figure 9:**
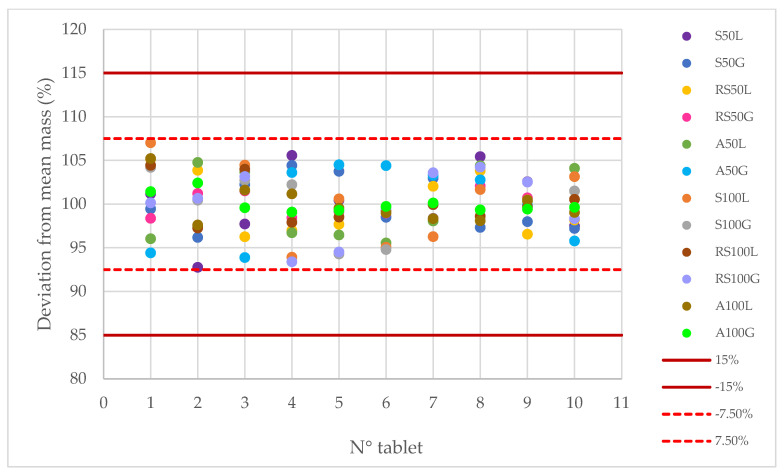
Mass variation of 3D-printed tablets.

Figure 9 represents the mass variation of the prepared 3D-printed tablet formulations. The permitted deviation in tablet mass is ±7.5% relative to the average mass, with pharmacopeial requirements allowing no more than two tablets to fall outside this range. At the same time, no individual tablet mass may deviate by more than ±15%. The obtained results indicate that all investigated formulations complied with the specified criteria.

#### 3.8.2. Uniformity of Content

**Figure 10:**
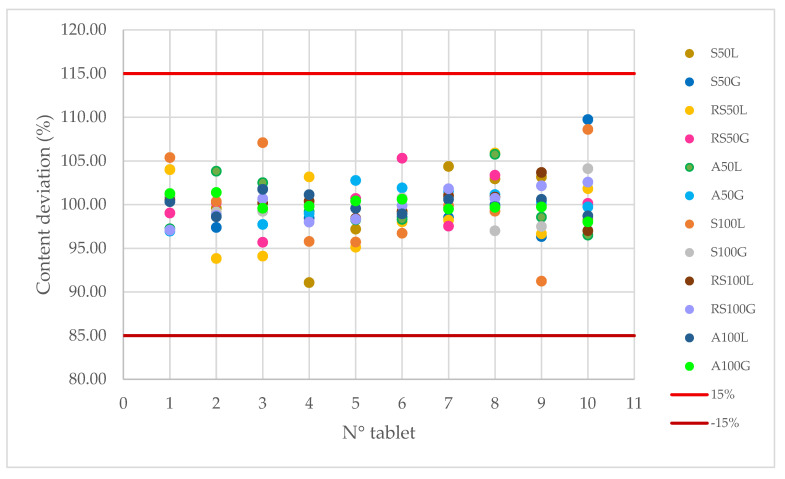
Content variation of PAC in 3D-printed tablet formulations.

The results of content uniformity testing expressed as a percentage of the mean PAC content are presented in Figure 10. According to pharmacopeial requirements, the content may deviate by ±15% from the mean content value, and all formulations complied with this criterion.

#### 3.8.3. Dimensions and Hardness of Printlets

The values of height, diameter, and hardness of the samples are presented in Appendix A, Table A1. Uniform diameter and height values of printlets prepared from the same type of filament indicate good reproducibility and precision of the printing process. The hardness values were similar among the formulations, which was expected considering the identical starting material.

#### 3.8.4. Drug Release Rate

**Figure 11:**
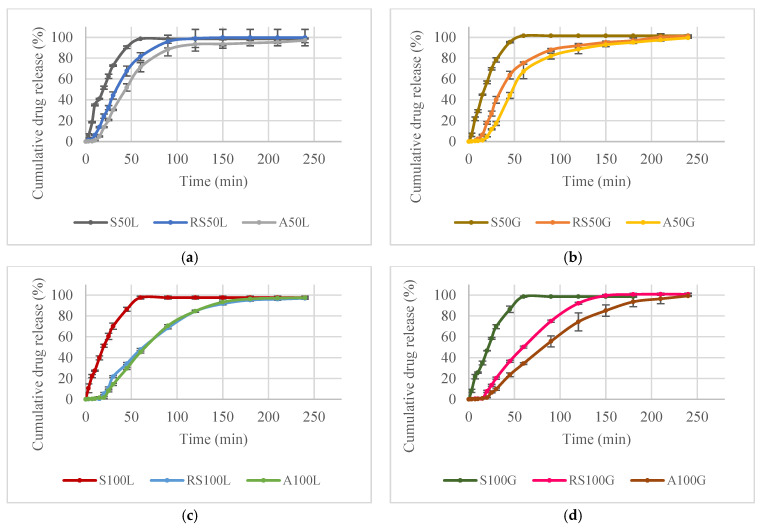
Release profiles of PAC from printlets: (**a**) S50L, RS50L and A50L; (**b**) S50G, RS50G and A50G; (**c**) S100L, RS100L and A100L; (**d**) S100G, RS100G and A100G.

The PAC release profiles of formulations with and without sodium alginate, with the same density and infill pattern, are presented in Figure 11a–d. Formulations containing a higher proportion of sorbitol (S50L, S50G, S100L and S100G) exhibited the fastest release of PAC, with S50G showing the highest release rate among all formulations. Additionally, a faster release profile was observed in formulations with decreased infill density. A more gradual release of PAC was observed for formulations containing less sorbitol. The most pronounced difference in release profiles among the sustained-release formulations was observed between RS100G (reduced sorbitol, 100% infill density and gyroid structure) and A100G (sodium alginate, 100% infill density and gyroid structure), while A100G showed the slowest release rate among all formulations, as shown in Figure 11d.

#### 3.8.5. Statistical Analysis

Statistical data processing was carried out using a model-independent method to compare formulations exhibiting similar release profiles and sustained-release behavior. The analysis was based on the values of the cumulative percentage of the released substance as a function of time, with the similarity of the profiles assessed using the difference factor (f_1_) and the similarity factor (f_2_). In order to examine the influence of individual factors on release kinetics, three types of comparisons were performed. The influence of infill density was analyzed by comparing formulations with 50% and 100% infill with the same composition and infill pattern (Table A2). Additionally, the influence of the composition of the formulation, i.e., the presence of sodium alginate, was examined by comparing formulations with and without this component and with the same structural parameters (Table 4). The influence of the infill pattern (lines in relation to the gyroid structure) was evaluated by comparing formulations of the same composition and infill density (Table A3).

Kinetic modeling of the dissolution profiles was performed for formulations S50G and A100G, representing the fastest and slowest drug release, respectively (Table A4). Among the evaluated models, the Weibull model provided the best fit for formulation S50G, as demonstrated by the highest R^2^ value (1.00), the lowest AIC value (66.93), and the highest MSC value (5.53). In contrast, formulation A100G was best described by the Logistic_2 model, which exhibited the highest R^2^ value (1.00), the lowest AIC value (77.95), and the highest MSC value (5.18). Higuchi and Korsmeyer–Peppas models showed considerably lower fitting performance.

## 4. Discussion

The results of the sieve analysis showed that PAC predominantly has a particle size distribution in the 180–355 µm fraction, while sorbitol exhibits a broader distribution ranging from <90 µm to 250 µm. On the other hand, PVA mainly consisted of fine particles smaller than 90 µm. Such a particle size distribution is considered suitable for the formation of pharmaceutical powders in HME, as it enables better particle packing, reduces mixture porosity, and contributes to improved flowability and content uniformity. In addition, the particle size of thermoplastic polymers such as PVA also affects the extrudability of the material, as well as its printability and interactions with the API [17,27]. In the study conducted by Saviano et al., moderately fine PVA particles (250–600 µm) showed better results in terms of drug content and printability compared with formulations containing larger particles of the thermoplastic polymer [27]. In the same study, the formulation containing particles smaller than 250 µm was not suitable for extrusion and printing. On the other hand, in the present study, the formulations contained PVA particles smaller than 90 µm and were successfully processed by HME. This difference may be related to the different experimental conditions and formulation preparation method, since the cited study used milled PVA filament, whereas the present study employed commercially available PVA powder recommended for HME applications. Therefore, the obtained results indicate that this particle size distribution was suitable for filament preparation. In addition, the particle size distributions of both commercially available excipients, PVA (Parteck^®^ MXP) and sorbitol (Parteck^®^ SI), were considered suitable for HME, as both are recommended by the manufacturer for this application. Furthermore, studies investigating various powder parameters, including particle size for HME, indicate an appropriate size range of <90 µm to 450 µm, leading to the conclusion that the sieve analysis results for PAC are also suitable [10,28].

The highest loss on drying was recorded for sodium alginate (12.08%), which is consistent with pharmacopeial requirements stating that the loss on drying for this substance should not exceed 15% [25]. This result can be attributed to its pronounced hygroscopic nature and ability to bind to moisture from the environment [29,30]. Sorbitol (0.73%) and PAC (0.95%) showed relatively low moisture content. Regarding the formulations, pre-drying values were 2.51% for F0, 2.46% for F1 and 2.39% for F4. Formulations F2 and F3 showed lower values, 2.05% and 1.86%, respectively. These results are important for the HME process and the production of filaments for FDM in 3D printing, as moisture significantly affects process quality. PVA, as a hydrophilic polymer, readily absorbs water, which can lead to changes in viscosity, bubble formation, and unstable extrusion. In this context, pre-drying of the formulations for 24 h at 40 °C represents a justified technological step, as reducing moisture content enables a more stable material flow through the extruder and reduces the risk of structural irregularities in the filament. Studies also highlight the importance of loss on drying testing in terms of reducing microbiological contamination of filaments and printlets. Water is a suitable medium for microbial growth; therefore, its removal reduces this risk. It has been reported that a mixture with 12% moisture achieved satisfactory extrusion performance, suggesting that our formulations meet the criteria of this test [31].

The extrusion temperature of pure PVA is 180 °C, and the addition of sorbitol as a plasticizer can reduce the extrusion temperature [32]. Grymonpré et al. reported in their study that the extrusion temperature of the PVA/sorbitol blend was 140 °C [33]. Based on this result, the extrusion mixture was investigated within a temperature range of 130–180 °C. Initially, the placebo formulation was used to optimize the extrusion parameters, which enabled the establishment of a stable processing window. Formulation F1 was processed at higher extrusion temperatures, whereas formulations F2–F4 required lower temperatures due to the presence of sorbitol.

Observed changes in filament F1 appearance at an extrusion temperature of 180 °C indicate possible deviations in the thermal behavior of the system compared to pure PVA. This may be related to the presence of the API, although the exact mechanism was not further investigated. Filaments of formulations F2, F3 and F4 exhibited pronounced flexibility, which can be attributed to the presence of sorbitol acting as a plasticizer in the PVA matrix [16]. Plasticizers reduce intermolecular forces between polymer chains, increasing their mobility and enabling filament bending without breaking [16]. In filaments containing sodium alginate, flexibility was maintained, indicating that the addition of this component at 5% did not significantly impair system elasticity. Furthermore, the presence of sodium alginate did not require changes in processing parameters during extrusion.

The spectrum of PAC showed its characteristic absorption pattern, including a broad O–H stretching region around 3400 cm^−1^ and C–H stretching bands near 2900 cm^−1^, while intense, defined stretching bands in the region 1750–1700 cm^−1^ can be attributed to the acetate ester and ketone groups of the steroid skeleton, followed by C=C stretching of the conjugated enone system near 1650–1600 cm^−1^ [34,35,36]. The region below 1500cm^−1^ corresponds to the fingerprint region. The obtained FTIR spectrum of PAC is in agreement with the spectral characteristics previously reported in the literature, confirming the identity of the drug substance [36].

The spectrum of PVA showed a broad absorption band in the region of 3600–3200 cm^−1^, which is attributed to O-H stretching vibrations, while the presence of a band around 2900 cm^−1^ indicates the asymmetric C-H stretching vibrations of the CH_2_ groups of this polymer. Additionally, the absorption band between 1600 and 1500 cm^−1^ is attributed to the C=C stretching vibration [34,35,37].

Sorbitol exhibited a broad band around 3400 cm^−1^, which was assigned to O–H stretching vibrations originating from the several hydroxyl groups present in this component. Stretching vibrations from C-H were attributed to the band near 2900 cm^−1^, whereas the peaks in the 1400–1000 cm^−1^ region were related to C–H bending and C–O stretching vibrations [34,35,38,39].

The FTIR spectrum of pure sodium alginate exhibited a broad absorption band attributed to O–H stretching vibrations [40]. The bands observed around 1600 cm^−1^ were assigned to the asymmetric stretching vibrations of O–C–O groups, whereas the band around 1400 cm^−1^ was attributed to C–OH deformation vibrations and symmetric O–C–O stretching vibrations [41]. The visible absorption bands around 1100–1000 cm^−1^ are associated with the polysaccharide backbone structure [41]. By analyzing the spectrum of the filaments, the dominance of the absorption bands of the matrix, PVA and sorbitol can be observed, which was expected considering the large mass fraction of these components, but also the low concentration of PAC (5% *w*/*w*).

Analysis of the filament spectra revealed the predominance of the absorption bands of the matrix components, PVA and sorbitol, which was expected considering the high content of these components and the low concentration of PAC (5% *w*/*w*). The relative intensities of the matrix absorption bands were consistent with the qualitative composition of the formulations. F2, containing the highest amount of sorbitol (45% *w*/*w*), exhibited the broadest and most intense O–H band, as well as the strongest C–O absorption in the fingerprint region, whereas formulation F3 (25% sorbitol and 70% PVA) showed absorption bands of lower intensity in these regions, in accordance with its lower sorbitol content. Formulation F4, which additionally contained sodium alginate (5% *w*/*w*), exhibited absorption in the 1650–1600 cm^−1^ region, characteristic of carboxylate groups. However, considering that the C=C stretching vibration of the PAC enone system is also located in this region, the individual contributions could not be distinguished. The PAC carbonyl bands (≈1750–1700 cm^−1^) were detected; however, their intensity was significantly reduced in the filament spectra, becoming progressively more noticeable from F2 to F4. The reduced intensity of these bands may be attributed to the low drug content (5% *w*/*w*), as well as to the extensive overlap with the absorption bands of PVA and sorbitol, which constitute the major fraction of the formulations [42]. Importantly, no new absorption bands were observed, nor were any shifts of the existing bands detected. This indicates good compatibility of the components and the absence of chemical interactions between them.

XRPD analysis of pure PAC revealed numerous sharp and intense Bragg reflections, with prominent peaks at approximately 2θ = 12.5° and 16°, together with additional well-defined reflections in the 10–22° region and several lower-intensity peaks up to approximately 40°. Such a diffraction pattern is highly characteristic of a material in the crystalline state. In contrast, the diffractograms of all three filament formulations (F2, F3 and F4) exhibited a broad amorphous halo with a maximum at approximately 2θ = 19–20°, characteristic of the amorphous PVA polymer matrix. The intense characteristic crystalline reflections of PAC were not observed in the filament diffractograms. Their disappearance in the filaments indicates the transformation of PAC from the crystalline to the amorphous state during the extrusion process. These findings are consistent with the formation of an amorphous solid dispersion, which is a characteristic feature of FDM 3D printing and drug formulations processed via the HME technique. The disruption of the crystalline structure of the API leads to the formation of an amorphous phase, thereby improving the apparent solubility and dissolution behavior of the drug [10,43,44].

The results shown in Figure 8 demonstrate successful tablet formation using FDM 3D printing. Tablets produced from filaments without sodium alginate had a white appearance, suggesting that the presence of this substance likely caused the color change. In all formulations, deposition lines are clearly visible, indicating a stable printing process with proper layer stacking. Visual inspection reveals a clear difference between tablets with higher infill density (S100L, S100G, RS100L, RS100G, A100L, and A100G) and those with lower infill density (S50L, S50G, RS50L, RS50G, A50L, and A50G). Additionally, tablets with 50% infill had a greater number of layers and consequently greater height compared to those with higher infill percentages, allowing us to achieve the required tablet mass and PAC dose. The front surfaces show more defined patterns, and printing appears more uniform and compact in formulations with higher infill density. Formulation S100L (Figure 8g) exhibited a more compact internal structure, with partial collapse of the lateral wall observed in the central region of the tablet. This phenomenon may be attributed to insufficient mechanical stability during material cooling, particularly in structures with higher material density, where heat accumulation can lead to localized deformation. In addition, the increased material mass within the internal region may have resulted in slower cooling rates and partial structural collapse [45]. In Figure 8i (RS100L tablet), thin strands of material are visible on the side, likely caused by material residue on the outer surface of the nozzle during non-extrusion movement [28]. In Figure 8j (RS100G tablet), partial separation of the internal structure from the outer wall is observed, which may be explained by weaker adhesion between these structures during FDM 3D printing [28].

For the mass variation test, 10 tablets from each formulation were analyzed. Based on these results, the criteria of Ph. Eur. 12 are met, which allow no more than two tablets to deviate outside ±7.5%, with none exceeding ±15% [25]. The smallest deviation from average mass was observed in formulation A100G. Minor deviations can be explained by characteristics of FDM 3D printing, where slight differences in filament diameter, material deposition, and extrusion rate fluctuations may affect final mass. Additionally, varying infill density can contribute to differences in internal material distribution.

Content uniformity was determined according to the Ph.Eur.12 requirements [25]. All contents are within ±15% and meet the requirements of pharmacopoeia. This result was expected because the PAC content in the filaments was also satisfactory, so the small variation in the PAC content is probably the result of the variation in tablet mass (higher tablet mass results in higher PAC content).

Dimensional measurements of the 3D-printed tablets showed minimal variation in height and diameter within the same formulation. The average tablet heights were 3.8 mm for S50L, 3.83 mm for S50G, 3.2 mm for RS50L and RS50G, 2.6 mm for A50L and A50G, 2.77 mm for formulation S100L and 2.82 mm for S100G. For tablet formulations RS100L, RS100G, A100L and A100G, a consistent tablet height of 2 mm was obtained. The low standard deviation within each group indicates a high degree of uniformity within the batches. Tablet diameters were approximately 10 mm across all samples, with average values ranging from 10.01 mm to 10.57 mm and low standard deviations. Similar findings can also be found in the literature, where a study on FDM 3D-printed theophylline tablets is reported [29]. In this study, it was determined that tablet dimensions, including diameter and thickness, largely correspond to the predefined values, with only minor deviations among individual samples. The reported standard deviations in printlet diameter of 0.1 mm indicate a low level of variability, which is expected given the use of commercially available PVA filament and the approach of producing hollow tablets that were subsequently filled with theophylline. In our study, slightly higher standard deviation values of diameter were recorded (below 0.25 mm), which may be attributed to differences in the manufacturing process; however, the obtained results still indicate good precision of the 3D printing of PAC tablets.

Regarding hardness testing, both the top (front) and lateral sides were evaluated. The lowest top hardness was observed for S100G (70.70), while the highest was recorded for A100G (97.90), with a gyroid structure, sodium alginate and 100% infill. The greatest lateral hardness was observed for A100L (96.90), and the lowest for S100L (75.30). Formulations with 50% infill without sodium alginate (S50L, S50G, RS50L and RS50G) showed higher side hardness compared to those with higher infill (S100L, S100G, RS100L and RS100G). Among the 100% infill formulations, those containing sodium alginate exhibited higher hardness for both the front and lateral surfaces compared to formulations without sodium alginate.

Dissolution profile figures show the influence of composition, internal structure, and infill percentage on release kinetics. The pronounced difference in the release profiles of formulations S50L, S50G, S100L, and S100G compared to all other formulations most likely results from the higher proportion of sorbitol in the mixture. Figure 11a (S50L, RS50L and A50L) shows nearly identical release rates between formulations RS50L and A50L, reaching a plateau at approximately 100 min, indicating minimal impact of sodium alginate. A similar trend is observed in Figure 11c between formulations RS100L and A100L. In Figure 11b (S50G, RS50G and A50G), S50G demonstrated a markedly faster release profile compared to RS50G and A50G. In contrast for the formulations RS50G and A50G, slight differences appear in early stages, but curves converge over time, indicating that the higher porosity at 50% filling partially compensates for the effect of the more complex geometry and the presence of sodium alginate. The most pronounced differences are seen in Figure 11d (S100G, RS100G and A100G), where the alginate-containing formulation shows significantly slower release, which can be attributed to the combined effect of the high infill density and the complex gyroid structure. This geometry increases the complexity of the diffusion pathways, while the presence of sodium alginate probably contributes to the formation of a gel layer, further slowing down the penetration of the medium and the diffusion of API.

The results shown in Table A2 indicate a significant influence of infill density on the release profile, independent of the composition and type of internal structure. In all observed pairs of formulations (RS50L–RS100L, RS50G–RS100G, A50L–A100L and A50G–A100G), the values of the difference factor (f_1_) are greater than 15, while the values of the similarity factor (f_2_) are less than 50, which clearly indicates different dissolution profiles. These results confirm that changing the infill density (50% vs. 100%) significantly affects the release kinetics. The observed accelerated release in formulations with a lower percentage of infill can be explained by the higher porosity of the system, which allows for easier penetration of the medium and faster diffusion of the API. Increasing the infill density leads to the formation of a denser structure with a smaller number of available diffusion paths, which results in a slowing down of the release process. The obtained results are in accordance with literature data [14,30]. In the work of Mandati et al., the influence of tablet shape and infill density on the release profile of FDM printed tablets was investigated, where it was shown that the infill density has a greater influence in relation to the shape, and a lower density of the infill leads to faster release [30].

In the case of formulations with a gyroid structure at 50% infill (RS50G–A50G), the similarity of the release profile was also observed, although the value of the similarity factor is close to the limit value (f_2_ ≈ 50), which indicates a slightly pronounced influence of sodium alginate in these conditions. However, in the case of formulations with a gyroid structure at 100% infill (RS100G–A100G), there is a deviation between the profiles, which is reflected in values of f_1_ greater than 15 and f_2_ less than 50. This behavior indicates that the combination of complex geometry and high density of the structure enhances the influence of sodium alginate on the dissolution process. The obtained results suggest that the influence of this component depends on the interaction between the geometry and the porosity of the system. While the linear structure ensures stable and predictable transport of the medium, the gyroid structure, especially at a high degree of infill, enables a more pronounced effect of this polymer on the release kinetics.

The results of the statistical analysis presented in Table 4 demonstrate the influence of the presence of sodium alginate on the dissolution rate of PAC at the same density and infill pattern. In formulations with a linear structure (RS50L–A50L and RS100L–A100L), a high degree of similarity between the release profiles was observed, as confirmed by difference factor values lower than 15 and similarity factor values higher than 50. These results imply that the addition of sodium alginate does not lead to significant changes in the release kinetics in systems with a simple, linear geometry.

According to the statistical analysis presented in Table A3, the change in the type of internal structure (linear versus gyroid), at the same degree of infill and composition, does not lead to significant differences in the dissolution profiles. In all analyzed cases (RS50L–RS50G, RS100L–RS100G, A50L–A50G and A100L–A100G), the values of f_1_ are less than 15, while the values of f_2_ are greater than 50, showing the similarity of the profiles. Such results suggest that the influence of the geometry of the structure on the release kinetics is less pronounced compared to the influence of the infill density. In one study, the effect of infill pattern (zigzag, cubic, tri-hexagonal and concentric) and wall thickness on the release of amlodipine from FDM 3D-printed tablets was systematically investigated, showing that different infill patterns, probably due to differences in porosity and internal structure, can significantly modify the release profile [46]. In contrast, in our work, linear and gyroid patterns were used without varying wall thickness, and no differences were observed between formulations with the same composition and infill percentage.

The superior fit of the *Weibull* model for formulation S50G suggests that the release of PAC from this formulation followed a complex release pattern that could not be described solely by diffusion-controlled mechanisms. Due to its flexibility, the *Weibull* model is frequently suitable for characterizing immediate-release and heterogeneous matrix systems, including FDM 3D-printed dosage forms. The rapid release observed for S50G may also be associated with the higher sorbitol content and lower infill density, which likely increased matrix porosity and facilitated faster penetration of the dissolution medium.

In contrast, the sustained-release formulation A100G was best fitted by the *Logistic_2* model, indicating a more complex and sigmoidal release behavior. This suggests the presence of multiple mechanisms controlling drug release, including diffusion through the polymer matrix and possible structural changes during dissolution. The higher infill density and more compact internal structure of A100G likely contributed to the sustained release profile by reducing medium penetration and slowing drug diffusion.

Overall, formulation A100G is identified as the most suitable system for achieving sustained release, due to the combination of gyroid structure, high infill density, and sodium alginate presence. Future studies may focus on increasing alginate content to further prolong release kinetics.

## 5. Conclusions

The application of HME technology enabled the production of filaments with PAC based on PVA, sorbitol and sodium alginate. Based on the visual evaluation of the filaments, it can be concluded that the composition of the formulation significantly affects their physical properties. The filament of formulation F1, which contained only PAC and PVA, showed an uneven structure with air bubbles and variations in thickness. In contrast, filaments F2, F3 and F4, which in addition to PAC contained other excipients, were uniform in thickness, without visible defects and similar in texture, which indicates optimally adjusted extrusion parameters. The uniform content of the API along the filament confirms the homogeneous distribution of the components in the polymer matrix, which is of key importance for precise and reproducible dosing in the further process of printing tablets.

FDM 3D printing allowed for the production of tablets with defined geometry and controlled internal structure. All formulations met the pharmacopeial requirements in terms of mass variation and content uniformity, while the tablet dimensions were in accordance with the predefined values, with minimal deviations. These results indicate the high precision, repeatability and reliability of FDM technology in the production of solid pharmaceutical forms.

This study demonstrated that the release rate of PAC from the tested formulations depends on the mutual interaction of composition, infill density and infill pattern. Among the investigated printing parameters, infill density showed the strongest influence on release kinetics. Moreover, higher sorbitol concentrations promoted faster drug release. The fastest release was observed for formulation S50G, indicating its suitability for immediate-release applications.

It was found that the presence of sodium alginate has no significant effect on the dissolution profile of formulations with a linear structure, regardless of the infill density. In contrast, sodium alginate formulations with a gyroid structure exhibited differences in the release rate at higher infill percentages, indicating a more pronounced influence of formulation composition in combination with a more complex geometry and higher infill density, likely due to the increased tortuosity of diffusion pathways and the gel-forming properties of sodium alginate. Based on the obtained results, the A100G formulation stands out as the most suitable for achieving the sustained release of the active substance.

Overall, it can be concluded that optimal design of modified release systems requires careful matching of composition and structural parameters. The obtained results confirm that the optimization of FDM 3D printing parameters is a useful tool for controlling and obtaining appropriate characteristics of pharmaceutical forms, which opens up the potential for the development of personalized therapeutic systems with a precisely defined API release profile and a dose adapted to the individual needs of each patient.

## Figures and Tables

**Table 1:** Composition of prepared formulations.

Component	Formulation Composition (%)				
	F0	F1	F2	F3	F4
PAC	-	5%	5%	5%	5%
PVA	50%	95%	50%	70%	70%
Sorbitol	50%	-	45%	25%	20%
Sodium alginate	-	-	-	-	5%

**Table 2:** Infill density, infill pattern, and predefined dimensions of printlets.

Formulation	Infill Density (%)	Infill Pattern	Predefined Dimensions (mm) Diameter × Height
S50L	50	Lines	10 × 2.6
S50G		Gyroid	
RS50L		Lines	10 × 3.8
RS50G		Gyroid	
A50L		Lines	10 × 3.2
A50G		Gyroid	
S100L	100	Lines	10 × 2
S100G		Gyroid	
RS100L		Lines	10 × 2.8
RS100G		Gyroid	
A100L		Lines	10 × 2
A100G		Gyroid	

**Table 3:** Determination of PAC content in powder formulations F1, F2, F3 and F4.

Formulation	Content (% of the Declared Value) ± SD *
F1	96.49 ± 1.11
F2	97.48 ± 1.89
F3	98.21 ± 1.46
F4	95.97 ± 0.18

* SD—standard deviation.

**Table 4:** Difference (f_1_) and similarity (f_2_) factors for the comparison of release profiles of formulations with and without sodium alginate, with the same infill density and pattern.

Formulations	f_1_	f_2_
RS50L-A50L	12.78	53.30
RS50G-A50G	12.88	50.15
RS100L-A100L	4.44	76.48
RS100G-A100G	16.82	49.28

**Figure A1:**
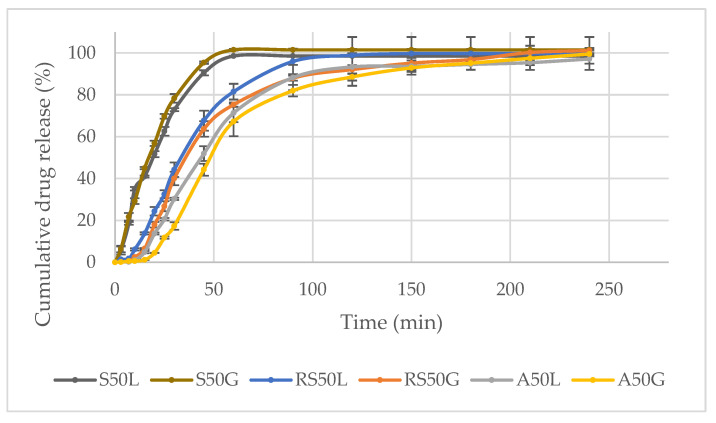
Release profile of PAC from printlets with 50% infill.

**Figure A2:**
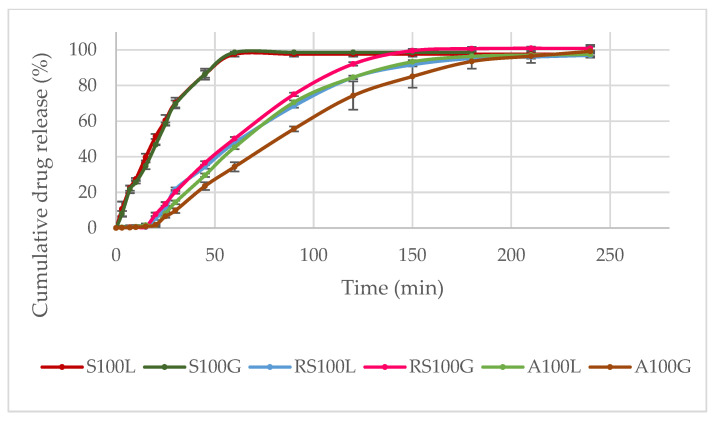
Release profile of PAC from printlets with 100% infill.

## Data Availability

The original contributions presented in this study are included in the article. Further inquiries can be directed to the corresponding author.

## Abbreviations

The following abbreviations are used in this manuscript:

API	Active Pharmaceutical Ingredient
FDM	Fused Deposition Modeling
HME	Hot Melt Extrusion
PAC	Prednisolone Acetate
PVA	Polyvinyl Alcohol
Ph. Eur. 12	European Pharmacopoeia 12th Edition

## Appendix A

**Table A1:** Values of printlet height, diameter, and hardness (front and lateral surfaces).

Height (mm)												
	**1**	**2**	**3**	**4**	**5**	**6**	**7**	**8**	**9**	**10**	**Mean**	**SD**
S50L	3.8	3.8	3.8	3.8	3.8	3.8	3.8	3.8	3.8	3.8	**3.8**	**0.00**
S50G	3.8	3.8	3.9	3.9	3.8	3.8	3.9	3.8	3.8	3.8	**3.83**	**0.05**
RS50L	3.2	3.2	3.2	3.2	3.2	3.2	3.2	3.2	3.2	3.2	**3.20**	**0.00**
RS50G	3.2	3.2	3.2	3.2	3.2	3.2	3.2	3.2	3.2	3.2	**3.20**	**0.00**
A50L	2.6	2.6	2.6	2.6	2.6	2.6	2.6	2.6	2.6	2.6	**2.60**	**0.00**
A50G	2.6	2.6	2.6	2.6	2.6	2.6	2.6	2.6	2.6	2.6	**2.60**	**0.00**
S100L	2.8	2.8	2.8	2.7	2.7	2.8	2.7	2.8	2.8	2.8	**2.77**	**0.05**
S100G	2.8	2.8	2.9	2.8	2.8	2.8	2.8	2.9	2.8	2.8	**2.82**	**0.04**
RS100L	2	2	2	2	2	2	2	2	2	2	**2.00**	**0.00**
RS100G	2	2	2	2	2	2	2	2	2	2	**2.00**	**0.00**
A100L	2	2	2	2	2	2	2	2	2	2	**2.00**	**0.00**
A100G	2	2	2	2	2	2	2	2	2	2	**2.00**	**0.00**
**Diameter (mm)**												
	**1**	**2**	**3**	**4**	**5**	**6**	**7**	**8**	**9**	**10**	**Mean**	**SD**
S50L	10	10	10	10.3	10	10	10	10	10	10	**10.03**	**0.09**
S50G	10	10	10.2	10.3	10.2	10	10.1	10	10	10	**10.08**	**0.11**
RS50L	10.4	10.5	10.2	10	10	10.2	10.3	10.4	10	10.1	**10.21**	**0.19**
RS50G	10	10.3	10.4	10.2	10.1	10.2	10.2	10	10.1	10	**10.15**	**0.14**
A50L	10.4	10.5	10.5	10.5	10.4	10.4	10.5	10.5	10.4	10.4	**10.45**	**0.05**
A50G	10.5	10.42	10.6	10.7	10.5	10.4	10.3	10.3	10.4	10.5	**10.46**	**0.13**
S100L	10	10	10.1	10	10	10	10	10	10	10.1	**10.02**	**0.04**
S100G	10	10	10.1	10	10	10	10	10	10	10	**10.01**	**0.03**
RS100L	10.5	10.4	10.7	10.6	10.5	10.4	10.3	10.5	10.4	10.3	**10.46**	**0.13**
RS100G	10.6	10.4	10.4	10.4	10.4	10.4	10.3	10.2	10.4	10.3	**10.38**	**0.10**
A100L	10	10.44	10.5	10.5	10.5	10.4	10	10.2	10.1	10.3	**10.29**	**0.21**
A100G	10.6	10.5	10.8	10.5	10.5	10.6	10.4	10.5	10.6	10.7	**10.57**	**0.12**
**Hardness of tablet front surface (shore A)**												
	**1**	**2**	**3**	**4**	**5**	**6**	**7**	**8**	**9**	**10**	**Mean**	**SD**
S50L	78	79	77	77	79	78	78	72	79	80	**77.70**	**2.21**
S50G	66	76	80	75	77	65	74	67	79	77	**73.60**	**5.54**
RS50L	90	98	86	95	90	94	92	93	96	95	**92.90**	**3.51**
RS50G	95	80	84	94	89	88	90	91	89	93	**89.30**	**4.57**
A50L	92	99	92	95	94	93	95	92	91	94	**93.70**	**2.31**
A50G	86	85	96	91	88	87	92	93	94	89	**90.10**	**3.67**
S100L	82	73	92	87	72	84	85	94	72	88	**82.90**	**8.10**
S100G	66	64	91	67	65	67	66	90	68	63	**70.70**	**10.54**
RS100L	94	86	88	92	90	91	88	89	95	94	**90.70**	**3.02**
RS100G	92	94	94	88	82	95	96	89	88	92	**91.00**	**4.27**
A100L	98	83	98	92	91	93	94	95	98	97	**93.90**	**4.63**
A100G	99	97	96	98	99	98	97	99	98	98	**97.90**	**0.99**
**Hardness of tablet lateral surface (shore A)**												
	**1**	**2**	**3**	**4**	**5**	**6**	**7**	**8**	**9**	**10**	**Mean**	**SD**
S50L	83	83	85	82	83	82	86	85	83	83	**83.50**	**1.35**
S50G	72	79	80	78	79	80	73	81	77	79	**77.80**	**3.01**
RS50L	90	98	84	96	88	89	97	95	92	93	**92.20**	**4.47**
RS50G	60	84	94	89	90	91	88	89	85	87	**85.70**	**9.48**
A50L	95	91	92	93	95	94	96	93	92	91	**93.20**	**1.75**
A50G	96	99	82	96	95	94	99	98	98	96	**95.30**	**4.97**
S100L	69	69	84	78	68	85	79	68	85	68	**75.30**	**7.63**
S100G	76	45	80	86	77	81	87	72	83	82	**76.90**	**12.10**
RS100L	96	78	90	90	85	86	92	91	89	84	**88.10**	**5.02**
RS100G	81	81	96	90	71	75	79	82	85	89	**82.90**	**7.39**
A100L	96	99	95	98	99	97	98	94	96	97	**96.90**	**1.66**
A100G	93	93	99	91	92	98	96	93	94	95	**94.40**	**2.59**

**Table A2:** Difference (f_1_) and similarity (f_2_) factors for comparing the release profiles of the tested formulations with different infill densities, with the same composition and infill pattern.

Formulations	f_1_	f_2_
RS50L-RS100L	24.53	37.20
RS50G-RS100G	15.59	45.36
A50L-A100L	16.35	46.06
A50G-A100G	17.26	44.10

**Table A3:** Difference (f_1_) and similarity (f_2_) factors for comparing the release profiles of the tested formulations with different infill patterns, but the same composition and infill density.

Formulations	f_1_	f_2_
RS50L-RS50G	7.36	65.17
RS100L-RS100G	7.28	68.63
A50L-A50G	8.46	61.75
A100L-A100G	9.86	59.89

**Table A4:** Parameters of the applied dissolution models for PAC release from 3D-printed tablets.

Formulation		Model Name and Parameters						
		Higuchi						
		R sqr	R sqr adj	AIC	MSC	kH		
S50G	mean	0.67	0.67	143.81	0.73	8.73		
	SD	0.01	0.01	0.60	0.04	0.04		
A100G	mean	0.84	0.84	134.24	1.66	5.89		
	SD	0.01	0.01	2.21	0.05	0.24		
MODEL		F = kH × t^0.5						
Formulation		Weibull						
		R sqr	R sqr adj	AIC	MSC	α	β	
S50G	mean	1.0	1.0	66.93	5.53	69.87	1.38	
	SD	0.0	0.0	7.10	0.45	20.23	0.09	
A100G	mean	0.99	0.99	91.43	4.34	21.76	6162.47	
	SD	0.00	0.00	7.75	0.53	2.14	0.09	
MODEL		F = 100 × {1 − Exp[−(t^β)/α]}						
Formulation		Korsmeyer–Peppas						
		R sqr	R sqr adj	AIC	MSC	kKP	n	
S50G	mean	-	-	-	-	-	-	
	SD	-	-	-	-	-	-	
A100G	mean	0.20	0.14	162.01	−0.07	0.01	1.79	
	SD	0.11	0.12	3.70	0.14	0.00	0.03	
MODEL		F = kKP × t^n						
Formulation		Hixson–Crowell						
		R sqr	R sqr adj	AIC	MSC	kHc		
S50G	mean	0.79	0.79	136.66	1.17	0.01		
	SD	0.01	0.01	0.66	0.05	0.00		
A100G	mean	0.95	0.95	115.26	2.85	0.00		
	SD	0.00	0.00	2.90	0.10	0.00		
MODEL		F = 100 × [1 − (1 − kHC × t)^3]						
Formulation		Logistic_1						
		R sqr	R sqr adj	AIC	MSC	α	β	
S50G	mean	0.99	0.99	95.67	3.73	5.46	4.62	
	SD	0.00	0.00	4.93	0.32	0.76	0.46	
A100G	mean	0.98	0.98	95.00	4.11	−11.32	6.18	
	SD	0.01	0.01	14.35	0.84	0.48	0.41	
MODEL		F = 100 × Exp[α + β × log(t)]/{1 + Exp[α + β × log(t)]}						
Formulation		Logistic_2						
		R sqr	R sqr adj	AIC	MSC	α	β	Fmax
S50G	mean	0.98	0.98	98.37	3.57	4.05	3.27	107.58
	SD	0.00	0.00	0.18	0.01	0.08	0.04	2.15
A100G	mean	1.00	1.00	77.95	5.18	−10.74	5.64	104.51
	SD	0.00	0.00	8.94	0.53	0.23	0.19	1.11
MODEL		F = Fmax × Exp[α + β × log(t)]/{1 + Exp[α + β × log(t)]}						
Formulation		Gompertz_1						
		R sqr	R sqr adj	AIC	MSC	α	β	
S50G	mean	0.97	0.96	109.33	2.88	23.68	3.08	
	SD	0.00	0.00	2.03	0.13	5.70	0.10	
A100G	mean	0.96	0.96	111.89	3.06	232.21	3.13	
	SD	0.02	0.02	9.40	0.51	74.67	0.29	
MODEL		F = 100 × Exp{−α × Exp[−β × log(t)]}						
Formulation		Gompertz_2						
		R sqr	R sqr adj	AIC	MSC	α	β	Fmax
S50G	mean	0.97	0.97	109.44	2.87	12.00	2.53	106.96
	SD	0.00	0.00	2.03	0.14	0.57	0.02	0.41
A100G	mean	0.98	0.97	106.43	3.40	113.26	2.65	115.26
	SD	0.01	0.01	7.96	0.41	29.14	0.24	8.65
MODEL		F = Fmax × Exp{−α × Exp[−β × log(t)]}						
